# Klassifikation aggressiver B-Zell-Lymphome: Neuigkeiten und offene Fragen

**DOI:** 10.1007/s00292-023-01187-4

**Published:** 2023-03-14

**Authors:** Andreas Rosenwald, Thomas Menter, Stefan Dirnhofer

**Affiliations:** 1 Institut für Pathologie, Universität Würzburg, Würzburg, Deutschland; 2 Pathologie, Institut für Medizinische Genetik und Pathologie, Universitätsspital Basel, Schönbeinstr. 40, 4031 Basel, Schweiz

**Keywords:** Burkitt-Lymphom, Diffuses großzelliges B‑Zell-Lymphom, Genrearrangement, Keimzentrum, Weltgesundheitsorganisation, Burkitt lymphoma, Diffuse large B‑cell lymphoma, Gene rearrangement, Germinal center, World Health Organization

## Abstract

Die 5. Edition der WHO-Klassifikation maligner Lymphome (WHO-HAEM5) und die Internationale Konsensus-Klassifikation (ICC) zeigen, was die Einteilung aggressiver B‑Zell-Lymphome angeht, erfreulicherweise nur wenige Unterschiede, die unseren diagnostischen Alltag wenig beeinflussen dürften. Auch die Neuerungen gegenüber der revidierten WHO-Klassifikation aus dem Jahr 2017 (WHO-HAEM4R) sind moderat. Sie betreffen meist geringfügige Namensänderungen einzelner Entitäten, Anpassung diagnostischer Kriterien oder eine Aufwertung „provisorischer“ zu „distinkten“ Entitäten. Die Definition des häufigsten aggressiven B‑Zell-Lymphoms, des diffus großzellige B‑Zell-Lymphom, nicht anderweitig klassifiziert (DLBCL, NOS), bleibt unverändert, eine Unterteilung in den Keimzentrumstyp bzw. Nicht-Keimzentrumstyp sehen beide Klassifikationen als wichtig an. Das DLBCL, NOS, muss im Rahmen der Routinediagnostik von anderen großzelligen B‑Zell-Lymphomen abgegrenzt werden. Dazu zählen das großzellige B‑Zell-Lymphom mit *IRF4*-Rearrangement, das in der ICC und WHO-HAEM5 zu einer definitiven Entität hochgestuft wurde, und das großzellige bzw. high-grade B‑Zell-Lymphom mit 11q-Aberration. Aggressive B‑Zell-Lymphome mit *MYC-* und *BCL2*-Rearrangement stellen biologisch eine homogene Gruppe dar und werden in beiden Klassifikationen als definitive Entitäten gelistet. Dies gilt nicht für die sehr heterogene Gruppe aggressiver Lymphome mit *MYC*- und *BCL6*-Rearrangements, welche von der ICC als provisorische Entität anerkannt werden, während die WHO-HAEM5 diese entweder unter den DLBCL, NOS oder den hochmalignen B‑Zell-Lymphomen, nicht anderweitig spezifiziert (HGBL, NOS), listet.

## Abstract (en)

The 5th edition of the WHO classification (WHO-HAEM5) and the International Consensus Classification (ICC) show a broad consensus in the categorization of aggressive, large B‑cell lymphomas with expected minor impact only on the daily diagnostic routine. The changes compared to the 2017 revised WHO-HAEM4R are moderate and include updated names of entities, sharpened diagnostic criteria, and upgrades from provisional to definite entities. The definition of the most common aggressive B‑cell lymphoma, diffuse large B‑cell lymphoma (DLBCL), not otherwise specified (NOS), remains unchanged, and both classifications strongly encourage subtyping into germinal center B‑like (GCB) or the activated B‑like (ABC or non-GCB) DLBCL. DLBCL, NOS, should be separated from other large B‑cell lymphomas including large B‑cell lymphoma with *IRF4* rearrangement (upgraded to a definite entity in both classifications) and large-cell/high-grade B‑cell lymphomas with 11q aberration. Aggressive B‑cell lymphomas with *MYC* and *BCL2* rearrangements form a molecularly distinct group and are listed as definite entities in both classifications. This is in contrast to the more heterogeneous group of aggressive B‑cell lymphomas with *MYC* and *BCL6* rearrangements that are recognized as a provisional entity in the ICC, while they fall into the DLBCL, NOS, or the HGBL, NOS, groups in the WHO-HAEM5.

Die 5. Edition der WHO-Klassifikation maligner Lymphome (WHO-HAEM5, [1]) und die Internationale Konsensus-Klassifikation (ICC, [3]) zeigen untereinander und auch im Vergleich zur revidierten 4. Fassung der WHO-Klassifikation (WHO-HAEM4R) in der Klassifikation aggressiver B‑Zell-Lymphome nur geringfügige Unterschiede, die auf unseren Routinealltag nur wenige Auswirkungen haben dürften. Keine Änderungen gibt es bei der häufigsten Entität, dem nicht weiter spezifizierten diffusen großzelligen B‑Zell-Lymphom (DLBCL, NOS). Eine exakte Auflistung der Entitäten großzelliger B‑Zell-Lymphome in der WHO-HAEM4R, WHO-HAEM5 und der ICC-Klassifikation findet sich in Tab. 1. Einige für die Diagnostik relevante Aspekte möchten wir im Folgenden etwas näher erläutern.

**Table 1:** 

WHO-HAEM4R	WHO-HAEM5	ICC
DLBCL, NOS (mit COO-Subtypen)	DLBCL, NOS (COO-Subtyp empfohlen)	DLBCL, NOS (mit COO-Subtyp)
Primäres DLBCL des Zentralnervensystems	Primäres DLBCL des Zentralnervensystems Davon abgegrenzt: Primäres DLBCL der Vitroretina	Primäres DLBCL des Zentralnervensystems
–	Primäres DLBCL des Hodens	Primäres DLBCL des Hodens
Primäres kutanes DLBCL, Beintyp	Primäres kutanes DLBCL, Beintyp	Primäres kutanes DLBCL, Beintyp
Intravaskuläres B‑Zell-Lymphom	Intravaskuläres B‑Zell-Lymphom	Intravaskuläres B‑Zell-Lymphom
–	Flüssigkeitsüberlastungsassoziiertes großzelliges B‑Zell-Lymphom	*HHV8- und EBV-negatives primäres ergussbasiertes Lymphom*
DLBCL, assoziiert mit chronischer Entzündung	DLBCL, assoziiert mit chronischer Entzündung	DLBCL, assoziiert mit chronischer Entzündung
Fibrinassoziiertes großzelliges B‑Zell-Lymphom (Subtyp des DLBCL, assoziiert mit chronischer Entzündung)	Fibrinassoziiertes großzelliges B‑Zell-Lymphom	Fibrinassoziiertes diffuses großzelliges B‑Zell-Lymphom (Subtyp des DLBCL, assoziiert mit chronischer Entzündung)
EBV-positives DLBCL, NOS	EBV-positives DLBCL	EBV-positives DLBCL, NOS
Lymphomatoide Granulomatose	Lymphomatoide Granulomatose	Lymphomatoide Granulomatose
EBV-positives mukokutanes Ulkus	EBV-positives mukokutanes Ulkus	EBV-positives mukokutanes Ulkus
–	–	*EBV-positive polymorphe B‑Zell-Lymphoproliferation, NOS*^a^
HHV8-positives DLBCL, NOS	KSHV/HHV8-positives DLBCL	KSHV/HHV8-positives DLBCL, NOS
Primäres Ergusslymphom	Primäres Ergusslymphom	Primäres Ergusslymphom
*Burkitt-ähnliches Lymphom mit 11q-Aberrationen*	High-grade B‑Zell-Lymphom mit 11q-Aberrationen	*Großzelliges B‑Zell-Lymphom mit 11q-Aberrationen*
*Großzelliges B‑Zell-Lymphom mit IRF4-Rearrangement*	Großzelliges B‑Zell-Lymphom mit IRF4-Rearrangement	Großzelliges B‑Zell-Lymphom mit IRF4-Rearrangement^b^
T‑Zell-/histiozytenreiches großzelliges B‑Zell-Lymphom	T‑Zell-/histiozytenreiches großzelliges B‑Zell-Lymphom	T‑Zell-/histiozytenreiches großzelliges B‑Zell-Lymphom
ALK-positives großzelliges B‑Zell-Lymphom	ALK-positives großzelliges B‑Zell-Lymphom	ALK-positives großzelliges B‑Zell-Lymphom
HGBL mit *MYC*- und *BCL2*-und/oder *BCL6-*Rearrangements	DLBCL/HGBL mit *MYC-* und *BCL2*-Rearrangements	HGBL mit *MYC-* und *BCL2-* Rearrangements *HGBL mit MYC- und BCL6- Rearrangements*
HGBL, NOS	HGBL, NOS	HGBL, NOS
Burkitt-Lymphom	Burkitt-Lymphom (Angabe, ob EBV-assoziiert)	Burkitt-Lymphom
Plasmablastisches Lymphom	Plasmablastisches Lymphom	Plasmablastisches Lymphom
Primäres mediastinales B‑Zell-Lymphom	Primäres mediastinales B‑Zell-Lymphom	Primäres mediastinales B‑Zell-Lymphom
Unklassifizierbares B‑Zell-Lymphom mit Merkmalen sowohl eines DLBCL als auch eines klassischen Hodgkin-Lymphoms	Mediastinales Grauzonenlymphom	Mediastinales Grauzonenlymphom

*COO* „cell of origin“, *DLBCL* diffuses großzelliges B‑Zell-Lymphom, *EBV* Epstein-Barr-Virus, *HGBL* high-grade B‑Zell-Lymphom,* NOS* nicht anderweitig spezifiziert

^a^ in der WHO-HAEM5 wird ein Teil dieser Fälle in der Kategorie der Lymphoproliferationen in Assoziation mit einer Immundefizienz/-dysregulation abgehandelt

^b^ in der ICC dem Bereich der follikulären Lymphome zugeordnet

## Diffuse großzellige B-Zell-Lymphome

Die Definition des häufigsten aggressiven B‑Zell-Lymphoms, des nicht weiter spezifizierten diffusen großzelligen B‑Zell-Lymphoms (DLBCL, NOS), hat sich in den aktuellen Klassifikationen nicht verändert. Ein Teil der morphologischen, immunhistochemischen und molekularen Heterogenität dieser Entität lässt sich weiterhin damit erklären, dass die neoplastische Blastenpopulation Differenzierungs- bzw. Reifungsprozesse von B‑Zellen im Keimzentrum bzw. nach dem Austritt aus dem Keimzentrum nachahmt. Im Rahmen der Pathogenese kommt es daher zum einen zu Mutationen von Genen, die für die Keimzentrumsentwicklung von Bedeutung sind (z. B. *EZH2*, *CREBBP*) oder zum anderen zu einer Abhängigkeit der Tumorzellen vom B‑Zell-Rezeptor- oder NF-kappaB-Signalweg im Postkeimzentrumssetting. Aus diesem Grund empfehlen sowohl ICC als auch WHO-HAEM5 für die Standarddiagnostik des DLBCL, NOS weiterhin die Angabe der Cell-of-origin(COO)-Klassifikation, i.e. den Keimzentrumstyp (GCB) oder den „aktivierten Typ“ (ABC bzw. Nicht-Keimzentrumstyp). Dies wird in der breiten Fläche weiterhin am ehesten mit einem immunhistochemischen Algorithmus, z. B. dem Hans-Klassifikator [10], erfolgen, da genexpressionsbasierte Tests für die Routinediagnostik meist nicht verfügbar sind. Die Unterteilung des DLBCL, NOS in einen Keimzentrumstyp und einen Nicht-Keimzentrumstyp hat weiterhin eine prognostische Bedeutung für die Patienten. Allerdings ergibt sich aus dieser Unterscheidung noch keine therapeutische Konsequenz, da in entsprechenden großen klinischen Studien kein Überlebensvorteil für Patienten mit DLBCL vom aktivierten B‑Zell-Typ durch Modifikationen der Therapie gezeigt werden konnte [6, 16]. Es gibt jedoch erste Hinweise darauf, dass jüngere Patienten mit einem DLBCL, NOS vom Nicht-Keimzentrumstyp von der zusätzlichen Gabe eines BTK-Inhibitors zum R‑CHOP-Regime profitieren könnten [22, 24].

In den letzten 4 Jahren haben 3 große genetische Studien mit Multi-Plattform-Technologien [4, 14, 20] zu einer Verfeinerung der Definition molekularer Subgruppen des DLBCL, NOS geführt (z. B. LymphGen-Algorithmus [19, 23]). ICC und WHO-HAEM5 sind sich aber darin einig, dass eine Anwendung solcher Algorithmen im Rahmen von aufwendigen molekularen Analysen derzeit im Alltag noch nicht sinnvoll ist und somit klinischen Studien oder wissenschaftlichen Programmen vorbehalten bleibt. In absehbarer Zukunft ist aber eine Subtypisierung des DLBCL aufgrund primär genetischer Unterschiede (Mutationen, Translokationen, strukturelle numerische Aberrationen) zu erwarten.

Die Standarddiagnostik des DLBCL, NOS im Routinesetting sollte daher gemäß ICC und WHO-HAEM5 neben der morphologischen Evaluation ein immunhistochemisches Markerpanel umfassen, das die differenzialdiagnostische Abgrenzung zu anderen, spezifischeren Entitäten der großzelligen B‑Zell-Lymphome erlaubt und eine Unterteilung in den Keimzentrumstyp bzw. Nicht-Keimzentrumstyp zulässt. Ferner sollte, wenn möglich, der Frage einer *MYC*-Translokation (und bei Nachweis eines Rearrangements mit konsekutiver Analyse der *BCL2*- und *BCL6*-Loci) nachgegangen werden, um eine Abgrenzung eines DLBCL, NOS von einem high-grade B‑Zell-Lymphom mit einem *MYC*- und *BCL2-* bzw. *BCL6-*Rearrangement zu ermöglichen (weitere Details siehe Abschn. „High-grade B‑Zell-Lymphome“).

Die WHO-HAEM5 fasst das primäre großzellige B‑Zell-Lymphom des ZNS, das primäre großzellige B‑Zell-Lymphom der Vitreoretina und das primäre großzellige B‑Zell-Lymphom des Hodens in der Gruppe der „großzelligen B‑Zell-Lymphome immunprivilegierter Lokalisationen“ zusammen. Bei diesen Lymphomen handelt es sich in der Regel um aggressive Tumore, die in Lokalisationen mit einem eigenen Immunmilieu entstehen und mit einer schlechten Prognose einhergehen. Immunphänotypisch gehören alle 3 Lymphome meist dem Nicht-Keimzentrumstyp an (CD10−, MUM1+) und zeichnen sich auf genetischer Ebene durch *MYD88*- und/oder*CD79b*-Mutationen aus. Ferner finden sich häufig genetische Alterationen in den MHC-Klasse-I/II-Genen sowie in *B2M* mit konsekutivem Verlust der Proteinexpression, welcher die Fähigkeiten der Tumorzellen zur Immunevasion fördert [12]. Aus diesen Gründen hat auch die ICC das primäre großzellige B‑Zell-Lymphom des Hodens als eigenständige Entität definiert. Allerdings verzichtet die ICC auf den Überbegriff der „großzelligen B‑Zell-Lymphome immunprivilegierter Lokalisationen“. Großzellige B‑Zell-Lymphome der Vitreoretina sind dort bei den primär großzelligen B‑Zell-Lymphomen des ZNS inkludiert.

Eine weitere Subklassifizierung der DLBCL-Familie betrifft die Einführung des flüssigkeitsüberlastungsassoziierten großzelligen B‑Zell-Lymphoms (WHO-HAEM5)/HHV8- und EBV-negativen primären ergussbasierten Lymphoms (ICC, provisorische Entität). Dieses Lymphom findet sich primär in Ergüssen und muss vom klassischen primären Ergusslymphom (PEL) abgegrenzt werden, das HHV8- und meist auch EBV-positiv und oft mit HIV assoziiert ist [2]. Im Vergleich zum klassischen PEL ist dieses Lymphom mit einer deutlich besseren Prognose assoziiert. Ob sich die strikte Definition der ICC mit Ausschluss EBV-positiver Fälle halten lässt, muss sich noch zeigen.

## Großzelliges B-Zell-Lymphom mit *IRF4*-Rearrangement

**Figure 1:**
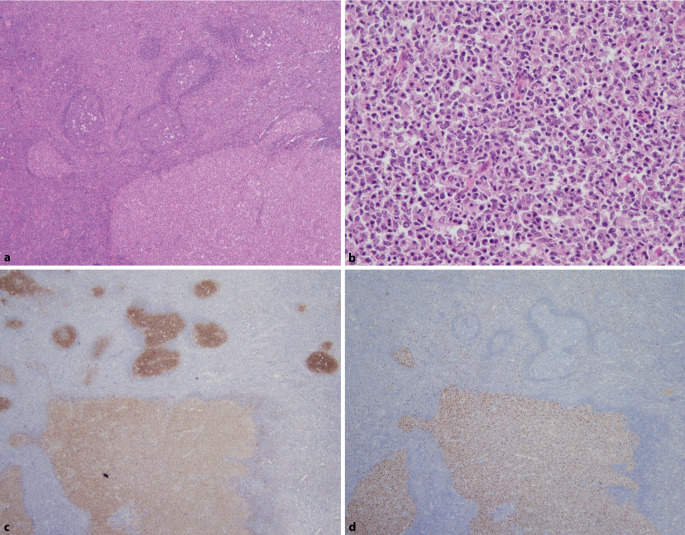

Diese Entität, die in der WHO-HAEM4R noch als provisorisch eingestuft war, wurde in der ICC und in der WHO-HAEM5 zu einer definitiven Entität hochgestuft. Zytologisch dominieren in dem Infiltrat relativ monomorphe, mittelgroße Blasten (Zentroblasten). Eine follikuläre Infiltratkomponente (morphologisch entsprechend einem follikulären Lymphom [FL] Grad 3B [ICC]/follikulärem großzelligem B‑Zell-Lymphom [WHO-HAEM5]) ist fast immer vorhanden. Häufig findet sich zusätzlich ein diffuses Infiltratmuster, nur gelegentlich wächst das Infiltrat ausschließlich diffus. Dieses Lymphom tritt meistens bei Kindern oder jungen Erwachsenen auf. Waldeyer-Rachenring und zervikale Lymphknoten sind in der Regel befallen. Aufgrund der fast immer vorhandenen follikulären Infiltratkomponente und des zumeist sehr indolenten Verlaufs wird dieses Lymphom in der ICC unter den follikulären Lymphomen gelistet, während es in der WHO-HAEM5 als Subtyp unter den großzelligen B‑Zell-Lymphomen fungiert. Immunhistochemisch zeigt sich fast immer eine kräftige Expression von MUM1/IRF4, meist bei starker Koexpression von BCL6. Auch CD10 wird in 50–60 % aller Fälle exprimiert (Abb. 1). Für die Diagnosestellung ist der Nachweis eines *IRF4*-Rearrangements (zumeist mit einem FISH-Ansatz) zwingend erforderlich. Molekularpathologisch finden sich häufig auch *IRF4*-Mutationen, was die Diagnose weiter untermauern kann. Der Nachweis eines *IRF4*-Rearrangements bei anderen aggressiven B‑Zell-Lymphomen, welche mit einem *BCL2*- oder einem *MYC*-Rearrangement assoziiert sind, ist ein unspezifischer Befund, welcher vor allem bei Erwachsenen auftritt [8, 18]. Dies erlaubt dann nicht die Diagnose eines großzelligen B‑Zell-Lymphoms mit *IRF4*-Rearrangement.

## Großzelliges bzw. high-grade B-Zell-Lymphom mit 11q-Aberration

**Figure 2:**
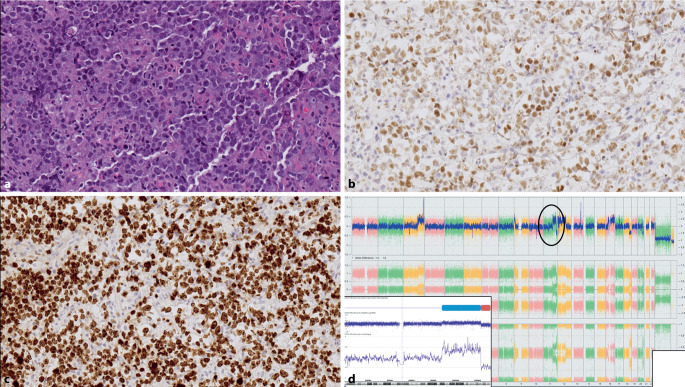

In der WHO-HAEM4R wurde erstmals die – provisorische – Entität des Burkitt-ähnlichen Lymphoms mit 11q Aberration beschrieben (Abb. 2). Die Namensgebung basierte auf der klinischen (Kinder bzw. junge Erwachsene), morphologischen und phänotypischen (CD10+/BCL6+/BCL2–) Ähnlichkeit mit dem Burkitt-Lymphom. Allerdings weisen diese Fälle kein *MYC*-Rearrangement auf. Sie zeichnen sich zytogenetisch per definitionem durch charakteristische Aberrationen auf 11q aus, im Speziellen einem Zugewinn in 11q23.2–23.3 sowie einem telomerischen Verlust in 11q24.1qter.

Weitere Studien zeigten dann, dass diese Fälle auch eine großzellige Morphologie aufweisen können und die Mutationslandschaft große Ähnlichkeiten mit konventionellen DLBCL vom GCB-Typ aufweist, wohingegen typische Burkitt-Mutationen (*ID3*/*TCF3* u. a.) fehlen [9, 21].

Daher haben sowohl ICC als auch WHO-HAEM5 die Bezeichnung dieses Lymphoms angepasst. In der ICC fungiert es weiterhin als provisorische Entität unter der Bezeichnung „großzelliges B‑Zell-Lymphom mit 11q-Aberration“, wogegen es in der WHO-HAEM5 als „high-grade B‑Zell-Lymphom mit 11q-Aberration“ bezeichnet wird.

## Burkitt-Lymphom

Sowohl die Definition als auch die diagnostischen Kriterien des Burkitt-Lymphoms (BL) bleiben unverändert in den beiden neuen Klassifikationen. Allerdings betont die WHO-HAEM5, dass die Bedeutung des EBV-Status (positiv vs. negativ) größere Relevanz bezüglich der Subtypisierung hat als die traditionelle Unterscheidung in endemische, sporadische und immundefizienzassoziierte Subtypen. Die ICC weist darauf hin, dass TdT-positive Fälle als B‑lymphoblastische Leukämie/Lymphom mit *MYC*-Rearrangement und nicht als BL klassifiziert werden sollten.

## High-grade B-Zell-Lymphome

Die WHO-HAEM4R beinhaltete folgende Subtypen als provisorische Entitäten: HGBL mit *MYC- *und *BCL2- *und/oder *BCL6-*Rearrangements („double-hit“ oder „triple-hit“) sowie high-grade B‑Zell-Lymphome (HGBL), NOS. Zwischenzeitlich haben verschiedene Studien gezeigt, dass HGBL mit *MYC- *und *BCL2-*Rearrangements eine homogene Gruppe darstellen. Sie weisen praktisch immer ein GCB-Genexpressionsprofil auf und haben häufig Mutationen, welche bei FL bzw. DLBCL vom GCB-Typ vorkommen, wie z. B. *CREBBP, BCL2, KMT2D, MYC, EZH2 *und *FOXO1. *Darüber hinaus lassen sich häufig *MYC*-Hotspotmutationen nachweisen [5, 7, 13].

Im Gegensatz dazu sind HGBL mit *MYC- *und *BCL6-*Rearrangements wesentlich heterogener [15, 17]. Es finden sich häufig extranodale Tumormanifestationen, das Genexpressionsprofil ist uneinheitlich und ebenso fehlt ein charakteristisches Mutationsspektrum. Aufgrund dieser Erkenntnisse haben sowohl die ICC als auch die WHO-HAEM5 „high-grade B‑Zell-Lymphome mit *MYC-* und *BCL2*-Rearrangements“ als eigenständige Entität anerkannt, wobei die WHO-HAEM5 die Bezeichnung „DLBCL/HGBL mit *MYC-* und *BCL2*-Rearrangement“ gewählt hat, um zum Ausdruck zu bringen, dass diese Fälle auch eine typische großzellige (DLBCL-ähnliche) Morphologie aufweisen können.

Beim HGBL mit *MYC- *und *BCL6-*Rearrangements (gemäß WHO-HAEM4R) ist das Vorgehen uneinheitlich. Die ICC erkennt diese weiterhin als provisorische Entität an. Eine zusätzliche Kategorie für die sog. Pseudo-double-hit-Lymphome (Fusion von *MYC* mit *BCL6*, was sich mittels FISH nicht von einer klassischen Translokation der beiden Gene unterscheiden lässt [11]) schafft die ICC nicht. Die WHO-HAEM5 hingegen schafft die gesonderte Einordnung der MYC- und BCL6-double-hit-Lymphome ab und klassifiziert diese Fälle gemäß der Morphologie entweder als Subtypen des DLBCL, NOS oder als HGBL, NOS.

HGBL, NOS wird definiert als B‑Zell-Lymphom mit blastoider oder Burkitt-ähnlicher Zytomorphologie, welches weder als DLBCL, NOS noch als Burkitt-Lymphom klassifiziert werden kann. Sowohl auf Ebene der Genexpression als auch auf Ebene der Mutationslandschaft handelt es sich um eine heterogene Gruppe. Letztendlich stellt es eine Ausschlussdiagnose dar. Sowohl die ICC als auch die WHO-HAEM5 weisen darauf hin, dass diese Diagnose nur sehr zurückhaltend gestellt werden sollte.

## Fazit für die Praxis

- Die Definition des diffusen großzelligen B‑Zell-Lymphoms, nicht anderweitig spezifiziert (DLBCL, NOS) bleibt in Internationaler Konsensus-Klassifikation (ICC) und 5. Edition der WHO-Klassifikation maligner Lymphome (WHO-HAEM5) unverändert. Eine Unterteilung in den Keimzentrumstyp bzw. Nicht-Keimzentrumstyp ist erforderlich bzw. empfohlen.
- Das großzellige B‑Zell-Lymphom mit *IRF4*-Rearrangement wird in beiden Klassifikationen zu einer definitiven Entität hochgestuft.
- Das „Burkitt-ähnliche Lymphom mit 11q-Aberration“ bekommt aufgrund des molekulargenetischen Profils eine neue Bezeichnung, nämlich „großzelliges B‑Zell-Lymphom mit 11q-Aberration“ (ICC) bzw. „high-grade B‑Zell-Lymphom mit 11q-Aberration“ (WHO-HAEM5). Die diagnostischen Kriterien bleiben jeweils unverändert.
- „Hochmaligne B‑Zell-Lymphome (HGBL) mit *MYC- *und *BCL2-*Rearrangements“ stellen molekular eine homogene Gruppe dar und werden daher in beiden Klassifikationen als eigenständige Entität definiert.
- „HGBL mit *MYC- *und *BCL6-*Rearrangements“ sind heterogen und werden nur von der ICC als provisorische Entität anerkannt. Die WHO-HAEM5 listet diese entweder unter den DLBCL, NOS oder den HGBL, NOS.
